# Coupling the recombineering to Cre-*lox* system enables simplified large-scale genome deletion in *Lactobacillus casei*

**DOI:** 10.1186/s12934-018-0872-4

**Published:** 2018-02-12

**Authors:** Yongping Xin, Tingting Guo, Yingli Mu, Jian Kong

**Affiliations:** State Key Laboratory of Microbial Technology, Shandong University, 27 Shanda Nanlu, Jinan, 250100 People’s Republic of China

**Keywords:** *Lactobacillus casei*, Large-scale genome deletion, genome engineering, Recombineering, Cre-*lox* system

## Abstract

**Background:**

*Lactobacillus casei* is widely used in the dairy and pharmaceutical industries and a promising candidate for use as cell factories. Recently, genome sequencing and functional genomics provide the possibility for reducing *L. casei* genome. However, it was still limited by the inefficient and laborious genome deletion methods.

**Results:**

Here, we proposed a genome minimization strategy based on LCABL_13040-50-60 recombineering and Cre-*lox* site-specific recombination system in *L. casei*. The LCABL_13040-50-60 recombineering system was used to introduce two *lox* sites (*lox66* and *lox71*) into 5′ and 3′ ends of the targeted region. Subsequently, the targeted region was excised by Cre recombinase. The robustness of the strategy was demonstrated by single-deletion of a nonessential ~ 39.3 kb or an important ~ 12.8 kb region and simultaneous deletion of two non-continuous genome regions (5.2 and 6.6 kb) with 100% efficiency. Furthermore, a cyclical application of this strategy generated a double-deletion mutant of which 1.68% of the chromosome was sequentially excised. Moreover, biological features (including growth rate, electroporation efficiency, cell morphology or heterologous protein productivity) of these mutants were characterized.

**Conclusions:**

To our knowledge, this strategy is the first instance of sequential deletion of large-scale genome regions in *L. casei*. We expected this efficient and inexpensive tool can help for rapid genome streamlining and generation restructured *L. casei* strains used as cell factories.

**Electronic supplementary material:**

The online version of this article (10.1186/s12934-018-0872-4) contains supplementary material, which is available to authorized users.

## Background

Lactic acid bacteria (LAB) are native to food-related habitats, including animal and vegetables niches and critical for the production of fermented beverages and foods [1]. In recent years, fast growing numbers of the whole genome sequences and functional genomics of lactic acid bacterial strains have provided an abundant of information for further understanding LAB, including their gene organization, their biological properties, and their ecological roles in animal or human health as well as their environmental interactions [2–5]. The whole genome sequences also offered the possibility for us to engineer LAB strains with custom-design and reduced genomes to serve as cell factories [6]. Therefore, the next widely significant breakthrough in genetic engineering is the ability to simply and efficiently reduce the genomes of LAB.

Of all the LAB stains, *Lactobacillus casei* plays key roles in dairy fermentations and pharmaceutical industries and is also the ideal cell factories for production of high-value metabolites [7–11]. However, the number of highly and satisfactorily engineered *L. casei* strains for biotechnological production purposes is still relatively low. Though the whole genome of *L. casei* BL23 has been sequenced and annotated in the GenBank database, it is still far from being well understood. By searching the genome of *L. casei* BL23 (GenBank: FM177140.1), almost 40% of its genes encoded for proteins with no assigned function [12]. Moreover, annotation of the genome relying on comparative genomics may result in inaccurate metabolic pathway or essential gene predictions [13, 14], limiting the processes on constructing novel strains with a markedly reduced genome that produce desired products efficiently. One of the best strategies for the veracity of *L. casei* BL23 functional genomics which is especially important for the whole genomics field is to simultaneously carry out one or a small number of genes deletion.

A high-efficiency genome editing tool in *L. casei* based on prophage-derived recombinases and Cre-*lox* system was established for construction of deletion mutants in our previous study [15]. This recombineering system consisted of a presumptive 5′–3′ exonuclease LCABL_13060, a ssDNA annealing protein LCABL_13050 and a predicted host nuclease inhibitor LCABL_13040 analogous to Exo, Beta and Gam, respectively. However, the hurdle for large-scale chromosomal deletion (> 6 kb) was the low efficiency of homologous recombination mediated by prophage-derived recombinases LCABL_13040-50-60. Therefore, construction of large-scale chromosomal deletion mutations was still based on a variety of conditional replication plasmids mediated by RecA dependent homologous recombination [16, 17]. These methods are useful but inefficient and laborious for large-scale region deletions, especially for the regions containing gene(s) impairing cell growth which are named important gene(s) in this study. Therefore, it is very instant to develop of a powerful and high-efficiency large-scale genome deletion method for identifying essential genetic information and engineering novel *L. casei* strains used for cell factories through experimental reduction of the genome to its minimal gene set in *L. casei*.

Large-scale genome engineering systems using for genome reduction have been described in bacteria [18–22]. Several of them are mainly based on the Cre-*loxP* site-specific recombination system of the bacteriophage P1 [18–20]. The Cre-*loxP* system was composed of two components: the 343 amino acid Cre recombinase facilitates the site-specific recombination and a 34 bp *loxP* site containing an asymmetric 8 bp core sequence and two 13 bp inverted repeats at which recombination takes place [23]. The Cre recombinase could excise any chromosomal region flanked by two *loxP* sites in which the 8 bp core sequence in same orientation, leaving one *loxP* site which limits the repeated use of the strategy [24, 25]. In order to efficiently and cyclically engineer large-scale genome, a pair of mutant *loxP* sites (*lox66* and *lox71*) was used for excision of the targeted region in the genome of *L. casei* BL23 strain.

Mobile genetic elements (MGEs) containing bacteriophages, transposable elements and genomic islands present in bacteria with continuous challenges to genomic stability, promoting evolution through horizontal gene transfer [26]. Comparative genome analysis showed that an insertion island (*LCABL_12890* to *LCABL_13480*) was exclusive for *L. casei* BL23, and the majority of genes were predicted to encode prophage-related proteins, suggesting the region is a prophage remnant [27]. In this study, the insertion island (*LCABL_12890* to *LCABL_13480*) was deleted coupling the *LCABL_13040*-*50*-*60* recombineering to Cre-*lox* system, and the properties of the resulting mutant strain (including growth rate and electroporation efficiency) were characterized. Moreover, the applicability or reproducibility of the method was demonstrated by the construction of a single deletion containing an important gene mutant or a double deletion mutant. This markerless large DNA deletion strategy represents a significant improvement to existing methods, and will accelerate the development of *L. casei* in genome streamlining and synthetic biology.

## Results

### Scheme for deletion of a large-scale genome region in *L. casei*

**Fig. 1:**
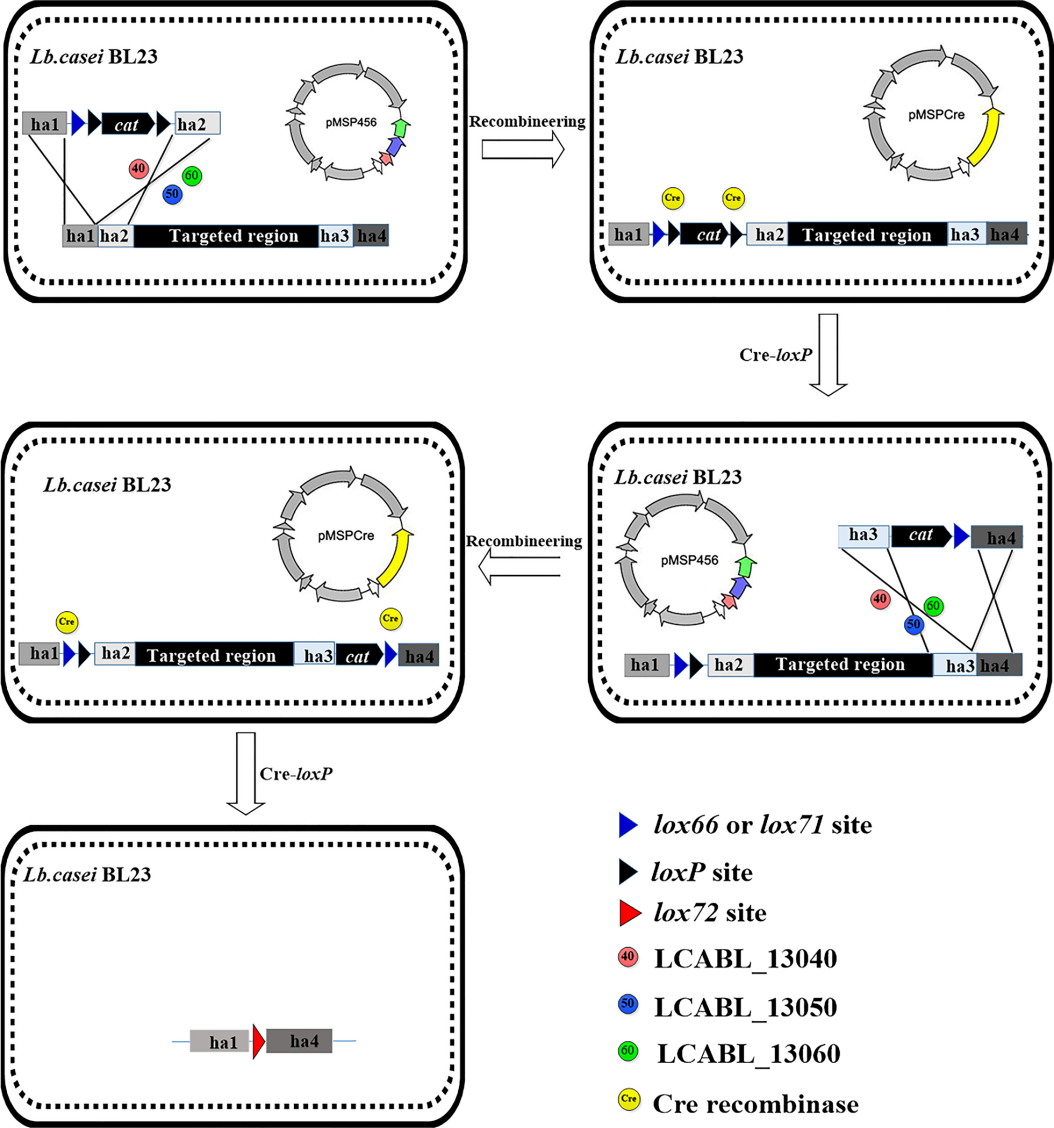
Scheme for markerless deletion of a large-scale genome region in *L. casei* BL23. Ha indicated four ~ 1 kb homology arms

To develop a large genome region deletion strategy in *L. casei*, the Cre-*lox* site-specific recombination system was used in this study (Fig. 1). However, the targeted large chromosomal DNA region needs to be introduced two parallel mutant *loxP* (*lox66* and *lox71*) sites (8 bp core sequence in same orientation) at 5′ and 3′ ends. Therefore, LCABL_13040-50-60 expression plasmid pMSP456 (Additional file 1: Figure S2A) and Cre recombinase expression plasmid pMSPcre (Additional file 1: Figure S2B) were employed, which the *LCABL_13040*-*50*-*60* and *cre* were both driven by an inducible P_nisA_ promoter, respectively.

Because the *LCABL_13040*-*50*-*60* recombineering system was not used for editing multiplex sites simultaneously, a two-step procedure was employed to sequentially introduce two parallel mutant *loxP* sites at 5′ and 3′ ends of the targeted region. Unfortunately, by two rounds of *LCABL_13040*-*50*-*60* recombineering tests, it was difficult to insert second mutant *loxP* site (*lox72*) into the 3′ end using the linear donor disruption cassette H-*cat*-*lox71*-H as the substrates with 2 × 5 or 4 × 5 mg/ml chloramphenicol selection. While the first mutant *loxP* site (*lox66*) was easy to insert into the 5′ end using h-*lox66*-*cat*-h as the substrates with 5 mg/ml chloramphenicol selection.

Therefore, an enhanced three-step strategy was explored to supply the inadequacy of the above two-step procedure. Initially, the first linear donor disruption cassette h-*lox66*-*loxP*-*cat*-*loxP*-h was used to insert the *lox66*-*loxP*-*cat*-*loxP* into the 5′ end of the targeted region by *LCABL_13040*-*50*-*60* recombineering. Secondly, the plasmid-free recombinant was transformed with pMSPcre, then induced to express Cre recombinase to excise the chloramphenicol resistance gene *cat* from the chromosome and leave *lox66*-*loxP* at the 5′ end of the targeted region. Finally, the *lox71* site resident in the second cassette h-*cat*-*lox71*-h was inserted into the 3′ end of the targeted region by another round of *LCABL_13040*-*50*-*60* recombineering. A large-scale genome region flanked with *lox*66 and *lox71* sites (8 bp core sequence in same orientation) will be excised in the presence of Cre recombinase.

### High-efficiency markerless deletion of a large-scale genome region in *L. casei*

**Fig. 2:**
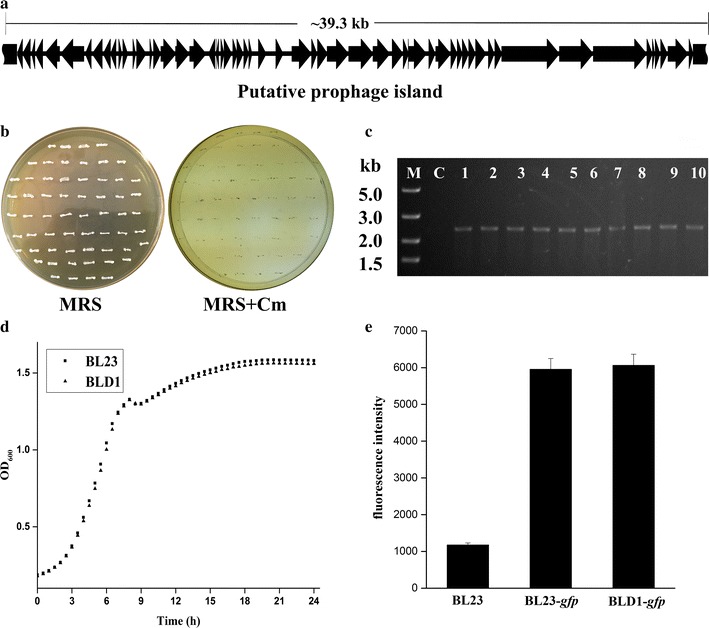
Deletion of large-scale genome region in *L. casei* BL23. **a** Layout and genetic context of the ~ 39.3 kb prophage island located on the chromosome. **b** Chloramphenicol resistance detection of BLD1 mutants. **c** Colony PCR results. M: DNA marker; C: wild-type strain; 1–10: chloramphenicol resistance colonies. **d** Growth of *L. casei* BL23 and the deletion strain *L. casei* BLD1 on MRS. **e** Comparison of fluorescence intensity of GFP in BL23, BL23-*gfp* and BLD1-*gfp*. Data represent the mean of three independent experiments

To test the scheme for markerless deletion of a large genome region, we initially searched the prophage-like genes in *L. casei* BL23 genome because prophages or prophage remnants may not always be necessary for the strain growth in the laboratory or industrial condition. Previously, a 38.9 kb genome region [27] which composed of main prophage remnants was identified and the genetic organization of this region was shown in Fig. 2a. This region contained 59 open reading frames (ORFs) and comprised approximately 1.27% of its genome. Among them, 19 genes have been annotated and ∼ 67.8% of the corresponding ORFs encoding proteins of unknown function. To assess the functions of this 38.9 kb DNA region, the mutants with the 38.9 kb genome region deletion were achieved by inducing with nisin to express Cre and subjecting to chloramphenicol resistance test. The sizes of homology regions ha1, ha2, ha3 and ha4 were 1187, 1114, 962 and 1121 bp in size, respectively. Colony PCR was subsequently used to further confirm the excision event with primer pair H1F and H2R. The expected size of PCR products amplified from the mutants should be ~ 2 kb (Fig. 2a). After induced with nisin for 12 h, all colonies tested were sensitivity to chloramphenicol (Fig. 2b). Results of colony PCR were coincident with the expected size using primers H1F and H4R (Fig. 2c). DNA sequencing further confirmed that gene *cat* has been taken out and a single *lox72* site has been successfully replaced by the 38.9 kb DNA fragment. Thus, these results clearly demonstrated that this strategy developed in this study could be used for construction of a 38.9 kb deletion mutant BLD1 with 100% efficiency.

Moreover, biological features of the mutant strain BLD1 (including growth rate, electroporation efficiency and heterologous protein productivity) were characterized. The growth profiles of strains were monitored by the optical density at 600 nm (OD_600_) of cells in MRS media. As shown in Fig. 2d, the deletion mutant BLD1 showed a slightly increased in both of growth rate and final OD_600_ compared to the wild-type strain BL23. Electroporation efficiencies of the mutant BLD1 and wild type strain BL23 were compared for two plasmids with different replication modes (pSec:Leiss:Nuc [28] and pTRKH2 [29]) and a temperature sensitive plasmid pG^+^Host9 [17] (Table 1). The electroporation efficiency of all the three tested plasmids for mutant BLD1 was approximately threefold higher than that for strain BL23. Moreover, the mutant BLD1 was evaluated as cell factories for heterologous protein production with the green fluorescent protein (GFP) as a reporter. Unexpectedly, the fluorescence intensity of the mutant BLD1-*gfp* was very similar to that of the strain BL23-*gfp* (Fig. 2e).

**Table 1:** The electroporation efficiency in BL23 and BLD1

Plasmids	Average number of transformants (CFU/µg plasmid DNA)	
	BL23	BLD1
pTRKH2	4.91 × 10^3^	1.64 × 10^4^
pG^+^host9	4.62 × 10^3^	1.39 × 10^4^
pSec:Leiss:Nuc	7.21 × 10^4^	1.77 × 10^5^

### Deletion of a large-scale genome region containing an important gene in *L. casei*

The high excision efficiency of Cre in *L. casei* BL23 encouraged us to test its activity in removal a large-scale genome region containing an important gene in *L. casei* BL23. Therefore, the 12.8 kb genome region which equipped with the putative important gene *galE* was selected as a target to be excised (Additional file 1: Figure S3A) by the above methods. The size of DNA fragment between two mutant *loxP* sites was around 12.8 kb. Twenty of the chloramphenicol sensitive colonies were picked and the excision of 12.8 kb DNA fragment was confirmed by PCR using primers H5F and H8R (Additional file 1: Figure S3B), the experimental data were coincident with the expected results. DNA sequencing of PCR products further proved the accurate excision (Biosune Company, Shanghai, China). Quantitative growth curves analysis indicated that the 12.8 kb deletion mutant BLD2 showed a decreased growth rate in MRS medium (Additional file 1: Figure S3C), suggesting that the deletion altered the strain’s phenotype. These results clearly demonstrated that this method could be used to delete a large-scale genome region containing an important gene in *L. casei* BL23.

### Simultaneous deletion of two non-contiguous regions in *L. casei*

**Fig. 3:**
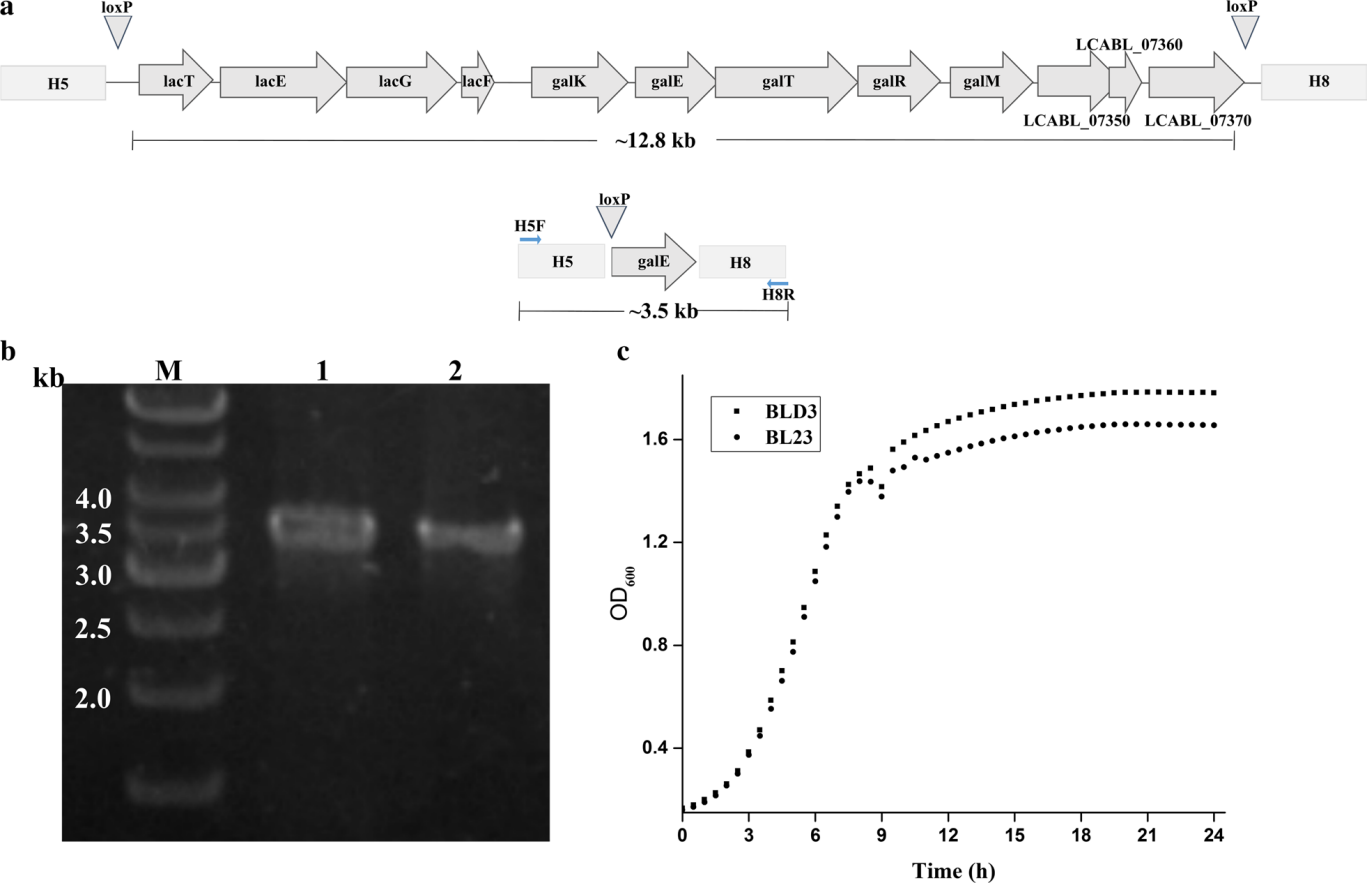
Simultaneously deletion of two non-continuous large-scale genome regions in *L. casei* BL23. **a** Layout and genetic context of the ~ 12.8 kb genome region and the sizes of PCR products amplified with primers H1F and H2R from BLD3 strains. **b** Colony PCR results. M: DNA marker; 1–2: random picked chloramphenicol resistance colonies. **c** Growth of *L. casei* BL23 and the deletion strain *L. casei* BLD3 on MRS

To investigate whether this procedure could be used for simultaneous deletion of two non-contiguous regions, the above 12.8 kb genome region was distributed into two DNA fragments by gene *galE* and the sizes of the two non-contiguous regions were 5.2 and 6.6 kb, respectively. For the simultaneous deletion of these two non-contiguous regions, we prepared the second disruption cassette h-*cat*-*lox71*-*galE*-h for replacing h-*cat*-*lox71*-h in the second round of *LCABL_13040*-*50*-*60* recombineering test. Subsequently, expression of the Cre recombinase would replace the 12.8 kb genome region with *lox72*-*galE*, resulting in the simultaneous deletion of these two non-contiguous regions, generating strain BLD3. Two randomly picked mutants were confirmed by PCR using primers H5F and H8R (Fig. 3a) and DNA sequencing. As shown in Fig. 3b, a mutant strain BLD3 with the targeted two non-contiguous region deletions was successfully constructed. Expectedly, only slight differences in growth rate was observed between the deletion strain BLD3 and the wild type strain BL23 (Fig. 3c).

### Second and successive deletion of large-scale genome regions in *L. casei*

**Fig. 4:**
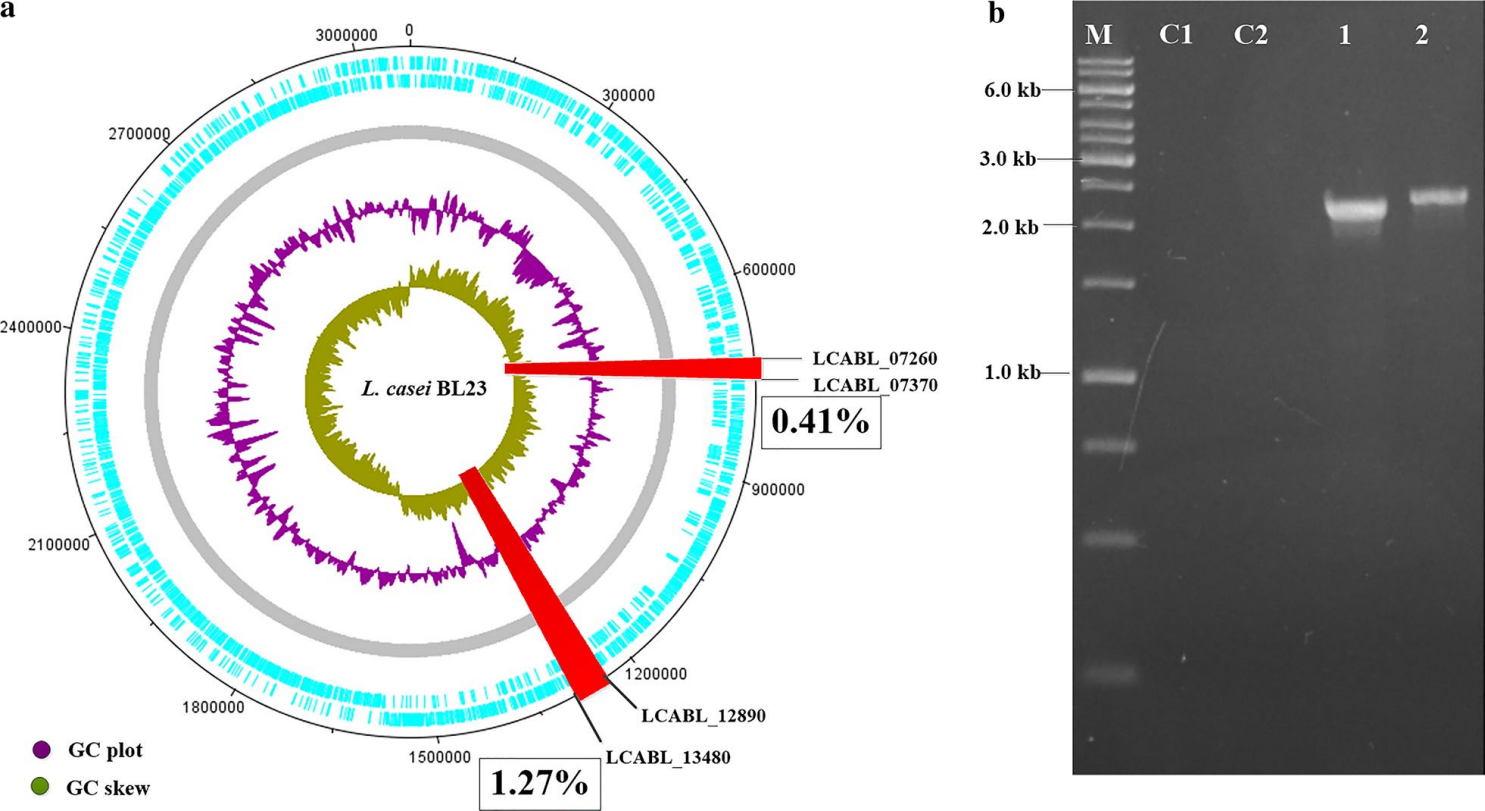
Sequential deletion of double large-scale genome regions in *L. casei* BL23. **a** Genomic deletions in the double-deletion mutants of *L. casei* BL23. The red boxes represent the 1.68% deleted regions. **b** Colony PCR results. M: DNA marker; C1: *L. casei* BL23 (12.8 kb); C2: *L. casei* BL23 (39.3 kb); 1: random picked chloramphenicol resistance colonies (12.8 kb); 2: random picked chloramphenicol resistance colonies (39.3 kb)

As a result of the first 39.3 kb genomic region deletion, the mutant strain BLD1 retains a single *lox72* site. To test whether this strategy has the capability of generating multiple deletions in *L. casei*, two mutant *loxP* sites aiming at excising the 12.8 kb region (Additional file 1: Figure S3A) was introduced into the mutant strain BLD1 by the same procedure (Fig. 1). After the recombination and excision step, markerless double-deletion of the two regions (1.68% of the genome size) was confirmed by PCR using pairs of primers (H1F/H4R and H5F/H8R) specific to the endpoints of the targeted region and sequencing (Fig. 4a, b). Therefore, this strategy described in this study was capable of creating successive chromosomal deletions in *L. casei* strain.

## Discussion

In recent years, large-scale genome engineering has been beneficial to the therapeutic and industrial applications, as well as a role in basic research into the origin and evolution of life and mechanisms of cellular metabolism [30–34]. However, it is still time-consuming and laborious to engineering a reduced *L. casei* genome. In this study, we proposed a novel genome engineering method for sequentially generating multiple markerless large-scale chromosomal deletions in *L. casei* BL23. To our knowledge, this is the first time to couple the recombineering to Cre-*loxP* system for simplified and programmable construction large-scale chromosomal deletions in *L. casei* BL23. First step in the strategy is to introduce two mutant *loxP* sites into both ends of the targeted region by *LCABL_13040*-*50*-*60* recombineering system and the Cre-*loxP* recombination system. Second step is to excise the large DNA by Cre recombinase.

The ~ 39.3 kb region (*LCABL_12890* to *LCABL_13480*) is a prophage remnant island for *L. casei* BL23 [27]. Prophage remnant islands, member of mobile genetic elements (MGEs), present in bacteria with continuous challenges to genomic stability [35]. However, our results strongly indicated that the deletion of this island increased the growth rate in MRS, suggesting that the reduced genome decrease the redundancy among genes and regulatory circuits [36]. This finding is in contrast to experiments targeting integrated mobile genetic elements, which resulted in a significantly longer generation time during log phase [35], possibly because this targeted region did not contain any core genes. Moreover, the length of the deleted region was larger than that in *Lc. lactis* [6, 37]. Considering the high-efficiency of deletion ~ 39.3 kb, larger-scale genomic regions could be excised for genome reduction using this strategy. However, our results showed that the heterologous GFP protein productivity of the engineered strain BLD1 with ~ 39.3 kb deletion was only slightly higher than that of the parental strain BL23, suggesting further studies should be done to identify and delete more nonessential DNA regions to reduce the genome and use the genome reduction strains as cell factories for enhanced heterologous protein expression.

Surprisingly, the efficiency of the ~ 12.8 kb genome region deletion which showed a strongly reduced growth rate when it was grown in a liquid medium containing glucose was nearly 100%. In *Lc. lactis* MG1363, deletion of *galE* gene would result in the reduced growth rate and long-chain phenotype [38]. Similarly, morphology analysis showed that the growth defect of the mutant cells was also be linked to formation of long chains of cells (Additional file 1: Figure S4). Our results also showed that mutant with the 12.8 kb deletion except for *galE* showed a very similar growth rates to the wild-type strain, suggesting that GalE catalyzing UDP-glucose to UDP-galactose plays a key role in the regulation of cell autolysis in *L. casei* BL23.

Previously, two regions separated by an essential gene should be excised independently in *L. casei*. Nevertheless, with this deletion strategy using a modified disruption cassette, simultaneous deletion of two noncontiguous genomic regions and excision of a genomic region that contains an essential gene could be performed rapidly. Additionally, this simultaneous noncontiguous deletion strategy also opens a new way for replacement deletion regions with available information and re-engineering large sections of the genome [20]. However, due to the low efficiency of DNA replacement mediated by prophage-derived recombinases LCABL_13040-50-60, deletion of targeted regions that contained an essential gene larger than 5.0 kb was not achieved in this study. Our results also showed that the *galKTRM* deletion strain *L. casei* BLD3 holds a very similar growth rate to *L. casei* BL23 in CDM medium supplemented with galactose (Additional file 1: Figure S5), suggesting that the major contributor to the catabolism of galactose was the tagatose-6-phosphate (T6P) pathway rather than the Leloir pathway in *L. casei* BL23 [39].

Currently, there is an increasing interest of using the CRISPR/Cas9 system for large-scale genome deletions in bacteria, including lactic acid bacteria [35]. However, the targeted regions were limited to be flanked by the homologous IS elements [35]. The application of the CRISPR/Cas9 or CRISPR-Cas9^D10A^ in *L. casei* for large-scale genome deletion (> 5 kb) has not been reported [40]. Instead, chromosomal deletions in *L. casei* have often been generated by using conditional replication plasmids [17, 41] or counterselection marker [16]. The new genome engineering strategy developed in this study has several practical advantages over those methods. First of all, both the efficiency of insertion a ~ 1 kb DNA fragment into the chromosomal locus mediated by *LCABL_13040*-*50*-*60* recombineering and excision the large-scale genome region between *lox66* and *lox71* sites were close to 100%. Therefore, the efficiency of this strategy was higher than those above methods in which only 50% of the double crossover colonies carrying the expected deletion in theory [16, 17, 41]. Secondly, this strategy does not require chemically defined or semi-defined media for counterselection [16], but only simply commercial MRS broth.

With this method developed here, the sequential introduced into the Δ39.3 and Δ12.8 kb double-deletion was performed, and the loss of the vector could be selected without erythromycin. Previously, it was successfully achieved the deletion of large regions of *Escherichia coli* K-12 chromosome using a combination of Cre-*loxP* and λ Red has been reported [42]. However, it was not used for successive deletion of large DNA segments due to the high-efficiency of site-specific recombination between two *loxP* sites (one *loxP* site from the first genomic excision and the new inserted *loxP* site) [42]. To address this problem, two mutant *loxP* sites were used in this study. Because the low-efficiency site-specific recombination between *lox72* and *loxP*, the cyclical genomic deletions could be achieved [43]. The repetition of the method on the double-deletion mutant as well as the establishment of further multiple-deletion mutants could allow further increases in the genome reduction beyond the 1.68% already obtained, leading towards determining a functional essential core of the genome under the specified conditions.

## Conclusions

In conclusion, we firstly proposed the targeted large-scale deletion method for genome streamline based on *LCABL_13040*-*50*-*60* recombineering and Cre-*loxP* site-specific recombination system in *L. casei*. With the modification of the linear donor disruption cassette, our strategy can be used for a variety of genome modifications. We also expected that this possible multiplexing method could provide a new way for rapid genome streamlining and generation restructured lactic acid bacteria strains served as cell factories with the development of the novel recombineering system in other lactic acid bacteria.

## Methods

### Bacterial strains, plasmids and growth conditions

Bacterial strains and plasmids used in this study are listed in Table 2. As a cloning host in this study, *E. coli* DH5α was grown in Luria–Bertani (LB) medium at 37 °C under agitation. Unless otherwise specified, lactobacilli and their derivatives used in this study were cultured in deMan Rogosa Sharpe (MRS) broth (Oxoid) at 37 °C under static conditions. When needed, the antibiotics were supplemented as followed: 5 μg/ml erythromycin or chloramphenicol for lactobacilli, 100 μg/ml ampicillin, 30 μg/ml kanamycin and 10 μg/ml chloramphenicol for *E. coli* DH5α, respectively.

**Table 2:** Plasmids and bacterial strains used in this study

Strain or plasmid	Characteristic(s)	Source
Strains		
*Escherichia coli* DH5α	F^−^ *supE44 ∆lacU169* Ф80*lacZ ∆M15 hsdR17 recA1 endA1 gyrA96 thi*-*1 relA1*	Novagen
*Lactobacillus casei* BL23	Derivative of *L. casei* ATCC 393 (pLZ15^−^)	[44]
Plasmids		
pG^+^host9	Erm^r^; temperature-sensitive vector	[17]
pUC19	Amp^r^; cloning vector	This study
pSec:Leiss:Nuc	pWV01 replicon; expresses Nuc under P_nisA_ control; Cm^r^	[28]
pET-28a	Kan^r^; cloning vector	This study
pTRKH2	Erm^r^; theta-replicating vector	[29]
pMSP456	Expression LCABL_13040-50-60 under P_nisA_ control	This study
pMSPcre	Expression Cre under P_nisA_ control	This study
pUCgalK	Source of fragment *loxP*-*cat*-*loxP*	[15]
pCD4033-*gfp*	Gene *gfp* as a reporter in the downstream of P_ldh_	[45]

### DNA manipulation

Plasmid Mini Kits (Omega) was used for *E. coli* plasmid DNA isolation and Gel Extraction Kits (Omega) or Cycle-Pure Kits (Omega) was used for the linear DNA purification. All the restriction enzymes, T4 DNA ligase and DNA polymerases used in this study were purchased from TaKaRa and strictly in accordance with the manufactural instructions for processing. PCR reactions for expressed purpose were generated with 2 × Primestar Max while PCR amplifications for screened purpose were performed by rTaq DNA polymerase.

### Construction of the linear donor disruption cassettes

All primers used for PCR amplification are listed in Table 3. To prepare of linear donor cassette (h1-*lox66*-*loxP*-*cat*-*loxP*-h2) for disruption the upstream of the ~ 39.3 kb region (Additional file 1: Figure S1), the chloramphenicol-resistance gene *cat* with its promoter region flanked by two *loxP* sites (*loxP*-*cat*-*loxP*) was obtained by general PCR from the vector pUCgalK [15] using primers catF1 and catR1. The upstream and downstream homology arms (h1 and h2) were PCR amplified from the chromosomal DNA of *Lb. casei* BL23 using primer pairs H1F/H1R and H2F/H2R. The restriction site SalI responsible for ligating to *loxP*-*cat*-*loxP* was introduced by the primers H2F. The two resultant fragments were spliced by an overlap extension PCR using primer pair H1F and H2R. The fusing product h1-*lox66*-h2 was digested with *Bgl*II and *Xho*I and ligated into the *Bgl*II and *Xho*I sites of pET-28a. The yielding vector pUD1 or *loxP*-*cat*-*loxP* were respectively digested with *Sal*I or *Xho*I and ligated to create pHA1. Finally, the linear donor disruption cassette h1-*lox66*-*loxP*-*cat*-*loxP*-h2 was generated by PCR from vector pHA1 using primers H1F and H2R.

**Table 3:** Oligonucleotide primers used in this study

Primer	Sequence (5′–3′)^a^	Restriction site
H1F	GAAGAAGATCATTTACTCGAG	*Xho*I
H1R	TACCGTTCGTATAGCATACATTATACGAAGTTATCAGAACGCAAATCTCTTCTA	
H2F	TGTATGCTATACGAACGGTAGTCGACTTACTTAATGCTATTCATTA	*Sal*I
H2R	TTAAGATCTACATCGAGTTCAGCAAGCTAA	*Bgl*II
H3F	ATATGTCGACATTACAGTTACAAGCCATATAC	*Sal*I
H3R	TCAATATTCTTCTCCGTCCC	
H4F	GGGACGGAGAAGAATATTGAAGATCTCACTGCAGTATATCAGGTAACAAAAAGT	*Bgl*II, *Sal*I
H4R	ACATGCATGCTTCCCTCTCCAAATATGCAC	*Sph*I
H5F	CGGGATCCAATAGCTCAGATTTTTAACAACA	*Bam*HI
H5R	TACCGTTCGTATAGCATACATTATACGAAGTTATTAATTTATCAAAAACCTTATTC	
H6R	CCCAAGCTTGTACATCATACTGTTCATGCC	*Hin*dIII
H6F	TGTATGCTATACGAACGGTACTCGAGAAGCCATCGCTGCTAACAAA	*Xho*I
H7F	TTTTGGCCAAGCTGGTTTT	
H7R	TTGTTTTTTCGCGGTACTGA	
H8F	TCAGTACCGCGAAAAAACAAGATCTCACACTCGAGTGTAGTCGATAAAAATT	*Bgl*II, *Xho*I
H8R	GCA ACATGCATGCAGACCATGAGACTATTTCAG	*Sph*I
PrF	TATAGCATACATTATACGAAGTTATCCAGCTAGGCCTAGTGTCCGT	
PrR	CGTCAATACCTCCTAATTGA	
galEF	TCAATTAGGAGGTATTGACGATGACAATTGCAGTTTTAGG	
galER	TTACTCGAGTCAATTCCGGTCACCAAATC	*Xho*I
catF1	CCGCTCGAGATAACTTCGTATAATGTATGCTATA	*Xho*I
catR1	CCGCTCGAGATAACTTCGTATAGCATACATTATA	*Xho*I
catF2	CCGCTCGAGATAACTTCGTATAGCATACATTATACGAACGGTACGAAAGTCGACGGCAATAG	*Xho*I
catR2	GGAAGATCTCTGTAATATAAAAACCTTCTTC	*Bgl*II

^a^The restriction sites in the primer sequences are underlined

To prepare of linear donor cassette (h3-*cat*-*lox71*-h4) for disruption the downstream of the ~ 39.3 kb region, the chloramphenicol-resistance gene *cat* with its promoter region flanked by a *lox71* sites (*cat*-*lox71*) was obtained by general PCR from the vector pUCgalK [15] using primers catF2 and catR2. The upstream and downstream homology arms (h3 and h4) were PCR amplified from the chromosomal DNA of *Lb. casei* BL23 using primer pairs H3F/H3R and H4F/H4R. The restriction sites *Bgl*II and *Sal*I responsible for ligating to *cat*-*lox71* was introduced by the primers H4F. The two resultant fragments were spliced by an overlap extension PCR using primer pair H3F and H4R. The fusing product h3–h4 was digested with *Sal*I and *Sph*I and ligated into the *Sph*I and *Xho*I sites of pET-28a. The yielding vector pUD2 or *cat*-*lox71* were respectively digested with *Sal*I or *Xho*I and *Bgl*II and ligated to create pHA2. Finally, the linear donor disruption cassette h3-*cat*-*lox71*-h4 was generated by PCR from vector pHA2 using primers H3F and H4R. The upstream or downstream linear donor disruption cassettes of the ~ 12.8 kb region was prepared by the similar method except for the skeleton plasmid pET-28a or pUC19.

### Integration of *lox* sites in *L. casei* BL23

Integration of *lox* sites in desired locus was achieved by the *LCABL_13040*-*50*-*60* recombineering. The *LCABL_13040*-*50*-*60* recombineering steps were carried out according to our previously study [15]. Briefly, *L. casei* strains harboring plasmid pMSP456 were grown in 5 ml MRS medium supplemented with 1% glycine and 0.75 M sorbital and cultured at 37 °C statically. Electrocompetent cells was prepared after induction LCABL_13040-50-60 recombinases expression by 5 ng/ml nisin at initial OD_600_ of 0.25–0.30 until OD_600_ of 0.60–0.65. The electroporation was performed utilizing a BioRad Genepulser and 2 mm electroporation cuvette after the mixture of linear donor dsDNA disruption cassette and electrocompetent cells was kept on ice for 10 min. After electroporation, 1 ml of SMRS was added to the cuvette and recovered at 37 °C for 1 h. Subsequently, the recover was plated on MRS plates containing chloramphenicol.

### Markerless deletion of a large-scale genomic region in *L. casei* BL23

The Cre-*lox* site-specific recombination system was used for markerless deletion of a selected regions. The steps were also carried out according to our previously study [15]. Briefly, plasmid-free mutants with two mutant *loxP* sites were grown and electrocompetent cells were prepared as described above. pMSPcre was introduced to the cells by electroporation and erythromycin resistant colonies were selected at 37 °C. The Cre recombinase expression was induced at OD_600_ of 0.4–0.8 with 10 ng/ml nisin for 24 h. After plated onto MRS plates, marker-free mutants were tested by PCR. Subsequently, the plasmid pMSPcre was cured at 37 °C without erythromycin for 24 h and streaked on MRS plates to obtain plasmid-free large DNA deletion mutants.

### Quantitative growth curves

*Lactobacillus casei* BL23 and its derivates were first grown at 37 °C in MRS for 24 h. Samples were harvested by centrifugation at 10,000*g* for 3 min. After being resuspended twice with MRS medium. 200 μl of bacterial suspension was subsequently loaded into a 96-well plate (Costar) in six wells per strain. The 96-well plate was incubated in a plate reader (Epoch2, BioTek) statically at 37 °C. Cell growth was detected at an absorbance of 600 nm, with readings obtained at 30 min intervals for 24. All liquid cultivations were conducted with six biological replicates and the growth curves were obtained for each well.

### Fluorescence assay

Fluorescence intensity of recombinant strains harboring the pCD4033-*gfp* [45] was determined according to our previous work [46]. Briefly, samples for measurement were taken out after 24 h and harvested by centrifugation at 10,000*g* for 3 min. After being resuspended twice with PBS buffer (pH7.4), 200 μl of bacterial suspension was transferred into a 96-well plate in which OD_600_ and fluorescence were read with excitation at 485 nm and emission at 528 nm using a Multi-Detection Microplate Reader, Synergy HT (BioTek). For each sample, three repetitions were performed with PBS as a blank.

## Additional file

**Additional file 1: Figure S1.** Strategies for obtaining linear donor disruption cassette H1-*lox66*-*loxP*-*cat*-*loxP*-H2. **Figure S2.** Structure of plasmids used in this study. Arrow heads show genes and their direction. (A) LCABL_13040-50-60-expression plasmid pMSP456. (B) Cre-expression plasmid pMSPCre. LCABL_13040-50-60 and Cre were transcribed from PnisA that is controlled by the product of nisin. **Figure S3.** Deletion of large-scale genome region containing important gene(s) in *L. casei* BL23. (A) Layout and genetic context of the ~ 12.8 kb genome region and the size of PCR products amplified with primers H1F and H2R from BLD2 strains. (B) Colony PCR results. M: DNA marker; C: wild-type strain; 1–20: chloramphenicol resistance colonies. (C) Growth of *L. casei* BL23 and the deletion strain *L. casei* BLD2 on MRS. **Figure S4.** Cellular morphologies of *L. casei* strains BL23 and BLD2. **Figure S5.** Growth of *L. casei* BL23 and the deletion strain *L. casei* BLD3 on CDM medium containing galactose.

## Abbreviations

LAB: lactic acid bacteria
MGEs: mobile genetic elements
