# Supplementary material for: Coupling the recombineering to Cre-lox system enables simplified large-scale genome deletion in Lactobacillus casei
Source: Microb Cell Fact. 2018 Feb 12;17:21. doi: 10.1186/s12934-018-0872-4 (PMC5808424; doi:10.1186/s12934-018-0872-4)
Supplement: Supplementary file 1 — Additional file 1: Figure S1. Strategies for obtaining linear donor disruption cassette H1-lox66-loxP-cat-loxP-H2. Figure S2. Structure of plasmids used in this study. Arrow heads show genes and their direction. (A) LCABL_13040-50-60-expression plasmid pMSP456. (B) Cre-expression plasmid pMSPCre. LCABL_13040-50-60 and Cre were transcribed from PnisA that is controlled by the product of nisin. Figure S3. Deletion of large-scale genome region containing important gene(s) in L. casei BL23. (A) Layout and genetic context of the ~ 12.8 kb genome region and the size of PCR products amplified with primers H1F and H2R from BLD2 strains. (B) Colony PCR results. M: DNA marker; C: wild-type strain; 1–20: chloramphenicol resistance colonies. (C) Growth of L. casei BL23 and the deletion strain L. casei BLD2 on MRS. Figure S4. Cellular morphologies of L. casei strains BL23 and BLD2. Figure S5. Growth of L. casei BL23 and the deletion strain L. casei BLD3 on CDM medium containing galactose. [file 12934_2018_872_MOESM1_ESM.pdf]

Manuscript title: Coupling the recombineering to Cre-*lox* system enables simplified large-scale genome deletion in *Lactobacillus casei*

Yongping Xin[1], Tingting Guo[1], Yingli Mu[1], Jian Kong[1]*

[1]State Key Laboratory of Microbial Technology, Shandong University, Jinan 250100, P. R. China

*To whom correspondence should be addressed. Tel: [+86 531 88362318]; Fax: [+86 531 88565234]; E-mail: [kongjian@sdu.edu.cn].

Present Address: [Jian Kong], State Key Laboratory of Microbial Technology, Shandong University, Jinan 250100, P. R. China

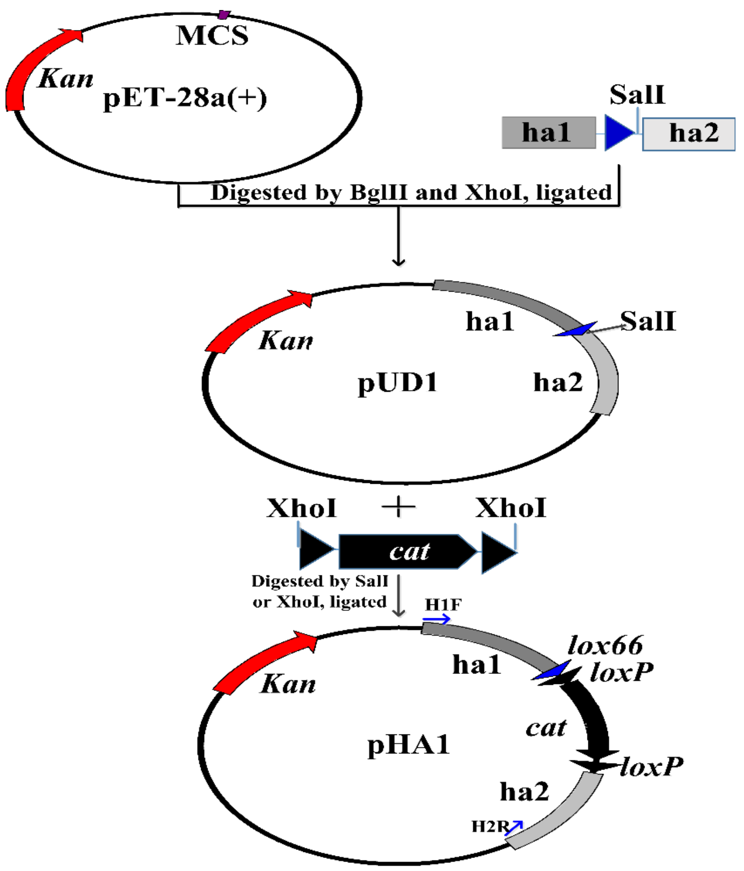

Figure S1. Strategies for obtaining linear donor disruption cassette H1-*lox66*-*loxP*-*cat*-*loxP*-H2.

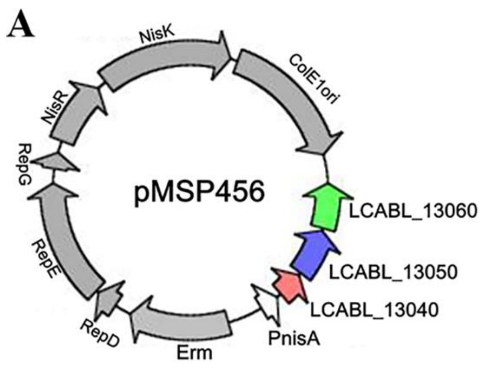

Figure S2. Structure of plasmids used in this study. Arrow heads show genes and their direction. (A) LCABL_13040-50-60-expression plasmid pMSP456. (B) Cre-expression plasmid pMSPCre. LCABL_13040-50-60 and Cre were transcribed from PnisA that is controlled by the product of nisin.

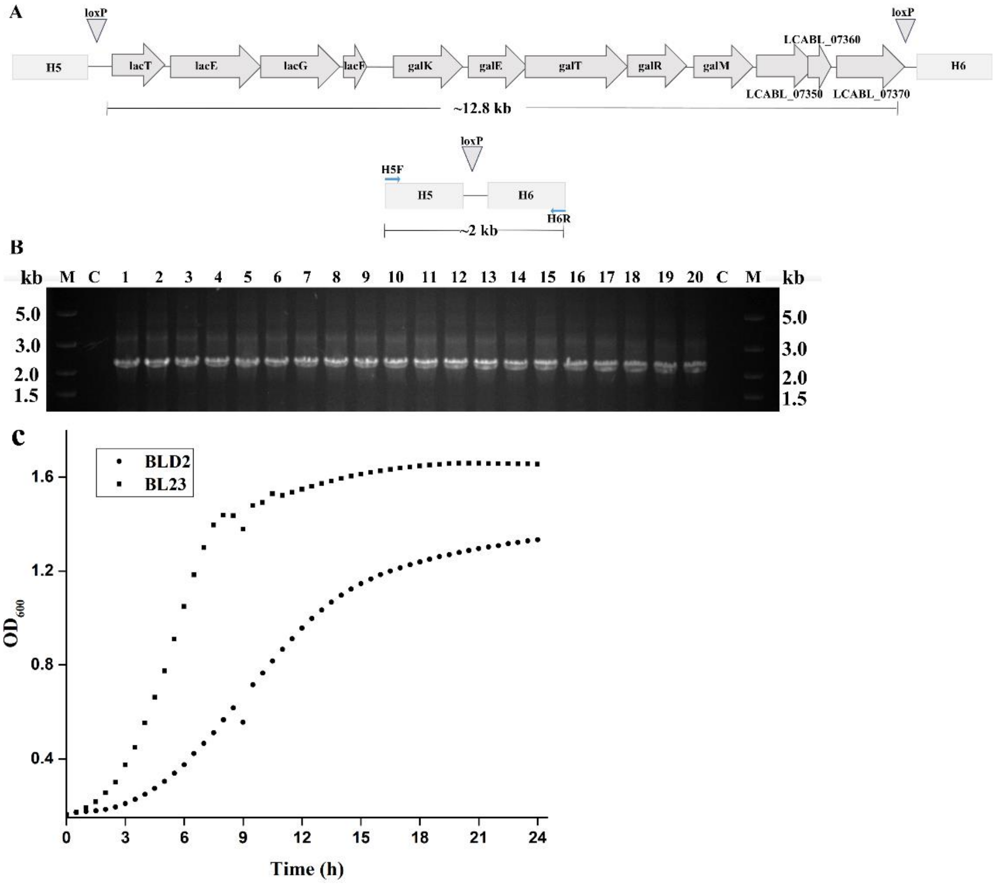

Figure S3. Deletion of large-scale genome region containing important gene(s) in *L. casei* BL23. (A) Layout and genetic context of the ~12.8 kb genome region and the size of PCR products amplified with primers H1F and H2R from BLD2 strains. (B) Colony PCR results. M: DNA marker; C: wild-type strain; 1–20: chloramphenicol resistance colonies. (C) Growth of *L. casei* BL23 and the deletion strain *L. casei* BLD2 on MRS.

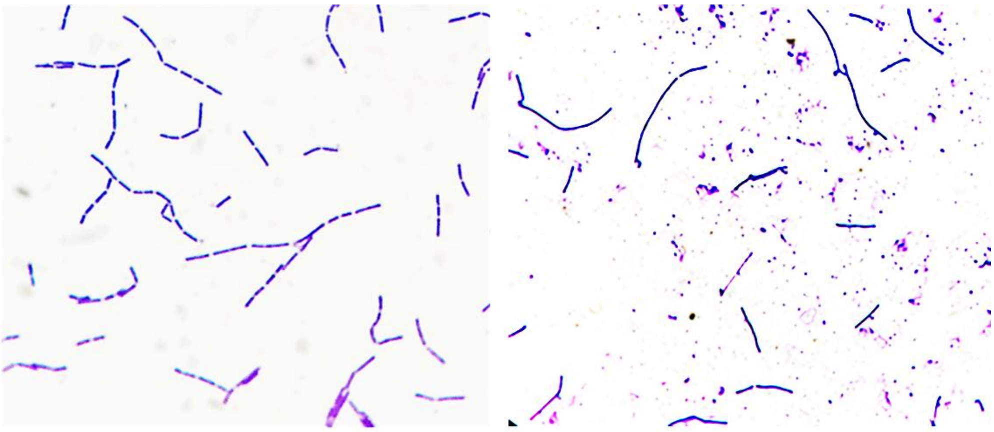

***Lactobacillus casei* BL23** ***Lactobacillus casei* BLD2**

Figure S4. Cellular morphologies of *L. casei* strains BL23 and BLD2.

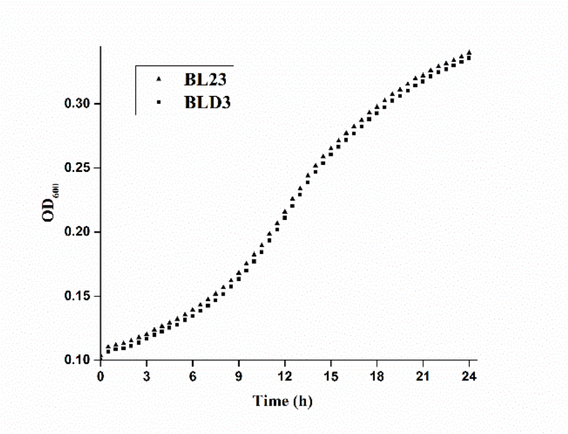

Figure S5. Growth of *L. casei* BL23 and the deletion strain *L. casei* BLD3 on CDM medium containing galactose.
